# Embracing Large Language Models for Medical Applications: Opportunities and Challenges

**DOI:** 10.7759/cureus.39305

**Published:** 2023-05-21

**Authors:** Mert Karabacak, Konstantinos Margetis

**Affiliations:** 1 Neurological Surgery, Mount Sinai Health System, New York, USA

**Keywords:** data privacy, ethical considerations, generative ai, chatgpt, multimodal learning, domain adaptation, reinforcement learning, transfer learning, artificial intelligence, large language models

## Abstract

Large language models (LLMs) have the potential to revolutionize the field of medicine by, among other applications, improving diagnostic accuracy and supporting clinical decision-making. However, the successful integration of LLMs in medicine requires addressing challenges and considerations specific to the medical domain. This viewpoint article provides a comprehensive overview of key aspects for the successful implementation of LLMs in medicine, including transfer learning, domain-specific fine-tuning, domain adaptation, reinforcement learning with expert input, dynamic training, interdisciplinary collaboration, education and training, evaluation metrics, clinical validation, ethical considerations, data privacy, and regulatory frameworks. By adopting a multifaceted approach and fostering interdisciplinary collaboration, LLMs can be developed, validated, and integrated into medical practice responsibly, effectively, and ethically, addressing the needs of various medical disciplines and diverse patient populations. Ultimately, this approach will ensure that LLMs enhance patient care and improve overall health outcomes for all.

## Editorial

Introduction

Large language models (LLMs) have been the focus of significant attention in the field of artificial intelligence (AI) in recent years. These models are trained on massive amounts of data and have demonstrated remarkable performance in natural language processing (NLP) tasks such as language generation, machine translation, and question-answering [1-3]. With the exponential growth of medical literature and the increasing availability of electronic health records (EHRs), LLMs are now poised to revolutionize medicine.

LLMs have the potential to transform medical practice in numerous ways, including improving diagnostic accuracy, predicting disease progression, and supporting clinical decision-making [4,5]. By analyzing large amounts of medical data, LLMs can rapidly develop specialized knowledge for different medical disciplines, such as radiology, pathology, and oncology [6-8]. They can be fine-tuned on domain-specific medical literature to ensure that they are up-to-date and relevant. They can be adapted to different languages and contexts, facilitating improved global access to medical knowledge and expertise.

However, integrating LLMs in medicine also presents significant challenges and limitations. The complexity of medical language and the diversity of medical contexts can make it difficult for LLMs to capture the nuances of clinical practice accurately. Furthermore, ensuring unbiased models and data privacy is crucial for fair and equitable healthcare. Collaboration among medical professionals, data scientists, ethicists, and policymakers is essential for comprehensive LLM development, addressing medical needs, challenges, and ethical implications. Therefore, this viewpoint article aims to provide a comprehensive overview of the potential benefits and challenges of using LLMs in medicine and identify key considerations for their successful implementation.

Transfer learning, domain-specific fine-tuning, and domain adaptation

Transfer learning is a powerful approach that allows LLMs to leverage pre-trained models as a starting point for further training and adaptation to medical domains [9]. By applying domain-specific fine-tuning, which involves training pre-trained LLMs on relevant medicine-specific data to perform well on tasks within the medical field, we can ensure up-to-date and relevant medical knowledge [10]. Prioritizing recent and highly cited articles can improve the model's performance in specific medical domains. This approach would allow for the rapid development of specialized LLMs that can address the unique needs of various medical disciplines.

Domain adaptation, closely related to domain-specific fine-tuning, is necessary for LLMs to function effectively in different medical domains, specialties, and languages. While domain-specific fine-tuning focuses on adjusting a model to perform well within a specific field, domain adaptation involves adapting a model trained in one domain to work effectively in a different but related domain without requiring extensive retraining [11]. Developing models that can adapt to various contexts ensures their applicability across diverse healthcare settings, benefiting both patients and practitioners. Moreover, the ability to adapt to different languages can help break down language barriers, facilitating improved global access to medical knowledge and expertise.

Alternative methods for adapting LLMs to medical domains, such as few-shot learning and zero-shot learning, can also be relevant in certain scenarios. Few-shot learning aims to train models to perform well on new tasks with very limited labeled data by leveraging knowledge learned from other tasks [12]. Zero-shot learning, on the other hand, focuses on training models to perform tasks without any labeled data for the target task, relying solely on knowledge learned from other tasks [13]. These approaches can be useful when domain-specific training data is scarce or unavailable, allowing LLMs to adapt to new medical domains more efficiently.

Several examples of LLMs fine-tuned for medical applications showcase the potential of transfer learning, domain adaptation, and alternative methods in this field. BioBERT, a pre-trained biomedical language representation model based on the BERT architecture, has been fine-tuned on large-scale biomedical corpora, including PubMed abstracts and PMC full-text articles, leading to significant improvements in biomedical NLP tasks such as named entity recognition, relation extraction, and question-answering [14]. ClinicalBERT, another domain-specific model, has been fine-tuned on the MIMIC-III dataset, which consists of EHRs from intensive care unit patients, demonstrating enhanced performance in clinical NLP tasks, including patient mortality prediction, de-identification, and diagnosis classification [15]. BlueBERT, also based on the BERT architecture and pre-trained on a large corpus of biomedical text data, has achieved state-of-the-art performance on various biomedical NLP tasks, including named entity recognition, relation extraction, and biomedical question-answering [16]. These examples highlight the success and potential of transfer learning, domain-specific fine-tuning, domain adaptation, and alternative methods in harnessing the power of LLMs for medical applications.

Reinforcement learning with expert input and dynamic training

Reinforcement learning is a type of machine learning where an agent learns to make decisions by interacting with an environment, receiving feedback in the form of rewards or penalties, and adjusting its actions accordingly [17]. In the case of developing LLMs for medicine, reinforcement learning with expert input is crucial for achieving accurate and unbiased models. Collaborating with medical experts who have agreed to a relevant declaration of principles would help grow trust in fairness, objectivity, and accuracy in model development. Expert feedback can help guide the model's learning process and enable a more nuanced understanding of complex medical concepts. This collaboration can lead to the creation of models that better understand and address the challenges faced by medical professionals in their daily practice.

Dynamic training is the continuous updating and training of a model to incorporate new data and knowledge, ensuring the model remains current and relevant [18]. For medicine-specific LLMs, it is essential to keep pace with the rapidly expanding medical knowledge. Continuously updating LLMs with new medical literature will allow them to remain current and adapt to emerging trends and discoveries. This approach is especially relevant for real-time applications, such as clinical decision support systems and telemedicine, where up-to-date information is crucial.

Collaboration and interdisciplinary approach

The successful implementation of LLMs in medicine requires collaboration between various stakeholders, including medical professionals, data scientists, ethicists, and policymakers [19]. An interdisciplinary approach ensures that LLMs are developed with a comprehensive understanding of medical needs and challenges, as well as the ethical, legal, and social implications of their use. Establishing partnerships between academia, industry, and healthcare providers can foster innovation and accelerate the translation of research findings into clinical practice.

In addition to involving these stakeholders, it is vital to include diverse perspectives, such as patients, caregivers, and representatives from different cultural backgrounds, in the development and evaluation of LLMs [20]. Incorporating these perspectives can help ensure that LLMs address the needs of diverse populations, leading to more equitable healthcare outcomes. Engaging with patient advocacy groups, community organizations, and other relevant parties can provide valuable insights into the unique challenges faced by different groups, enabling LLM developers to create models that are tailored to their specific needs. Furthermore, involving diverse perspectives can help identify potential biases and unintended consequences in LLM outputs, promoting fairness and inclusivity in the development and application of these technologies.

Education and training

Education and training are essential for the effective integration of LLMs into medical practice. As LLMs become more prevalent in healthcare, medical professionals need to understand their capabilities and limitations and how to use them effectively in their clinical practice. Medical curricula should incorporate fundamental concepts of AI, machine learning, and LLMs, providing future practitioners with the necessary knowledge and skills to work with these technologies. This training should include an understanding of how LLMs work, how they can be adapted and fine-tuned to specific medical domains, and how to interpret the model's outputs. Medical students should also receive training in data ethics, privacy, and security to ensure they use LLMs in an ethical and responsible manner.

Continued professional development programs should be available for current healthcare providers to ensure they remain competent in using the latest advancements in LLMs and other AI technologies. These programs should be tailored to the specific needs of different healthcare professionals, such as physicians, nurses, and other allied health professionals. The training should include hands-on experience with LLMs, such as how to fine-tune a pre-trained model for a specific medical application or how to interpret the model's predictions. Additionally, the training should address the challenges and limitations of LLMs in medicine, such as potential biases, privacy concerns, and ethical considerations.

Moreover, the training should also cover how to integrate LLMs into the clinical workflow, how to communicate LLM-generated outputs to patients, and how to collaborate with data scientists to fine-tune LLMs to specific clinical needs. It is also important to involve patients and caregivers in the training and education process, as they can provide valuable feedback on the usefulness and usability of LLM-generated outputs in clinical decision-making. Overall, comprehensive education and training programs can ensure that healthcare professionals are equipped with the necessary skills and knowledge to effectively and responsibly use LLMs in clinical practice.

Evaluation metrics, benchmarks, and clinical validation

Establishing robust evaluation metrics and benchmarks is essential for assessing the performance of LLMs in medical applications [21]. Traditional evaluation methods may not be sufficient as they may not account for the specific challenges and requirements of the medical domain. Developing new evaluation standards that consider both the technical performance and real-world utility of these models is crucial.

Clinical validation, in collaboration with medical professionals, is necessary to assess the real-world utility of LLMs. Rigorous evaluation of their performance in clinical settings can help identify areas for improvement and ensure that the models are beneficial to patients and healthcare providers. The validation process should include diverse clinical scenarios and patient populations to ensure that the models are capable of addressing a wide range of medical challenges. The validation process should aim at both the application process and the content creation of the LLMs in medicine.

Challenges and limitations of LLMs in medicine

While LLMs have the potential to revolutionize medical practice, it is essential to address their challenges and limitations to ensure their safe and effective use. One significant concern is the risk of over-reliance on AI technologies, leading to reduced human input in critical decision-making processes. In particular, medical professionals must be cautious about interpreting AI-generated outputs and validating them against their expertise and context. The development of LLMs should focus on augmenting human expertise rather than replacing it, ensuring that medical professionals retain a central role in patient care.

Another challenge is the potential for LLMs to inadvertently generate misleading or incorrect information, which could have severe consequences in healthcare settings. Ensuring the accuracy and reliability of LLM-generated outputs is crucial, as errors could lead to incorrect diagnoses, inappropriate treatments, or other negative patient outcomes. To mitigate these risks, rigorous validation processes, continuous monitoring, and collaboration with medical experts are essential. Furthermore, developing explainable AI techniques can help medical professionals better understand the underlying reasoning behind the LLM-generated outputs, enabling them to identify and address potential issues more effectively.

Additionally, implementing LLMs in healthcare settings comes with significant cost and resource implications. Developing, training, and maintaining LLMs require substantial computational resources, which can be a barrier to widespread adoption, especially in low-resource settings. Consequently, it is crucial to explore alternative solutions that address these challenges. One possibility is the development of more efficient models that require less computational power while maintaining high performance. Another option is leveraging cloud-based resources to allow healthcare providers to access AI capabilities without investing in expensive hardware and infrastructure. Moreover, exploring collaborations between the public and private sectors could help distribute the costs and resources needed for LLM implementation more equitably, ensuring that these transformative technologies become accessible to a broader range of healthcare settings. By addressing these challenges and limitations, LLMs can be integrated more safely and effectively into medical practice, maximizing their potential to improve healthcare outcomes for diverse populations.

Ethical considerations, data privacy, and regulatory framework

Ethical considerations are paramount when implementing LLMs in medicine. To ensure unbiased models and mitigate potential biases, it is crucial to focus on fairness and equitable healthcare. This can be achieved through fairness-aware machine learning, a subfield that aims to develop algorithms and models that consider fairness and minimize biases by accounting for the potential disparate impact on different demographic groups [22]. Counterfactual fairness, a criterion that evaluates the fairness of a model by comparing its decisions for an individual with hypothetical alternative decisions it would have made if the individual belonged to a different group, is another important aspect of achieving fair outcomes [23]. This approach helps ensure that models treat individuals consistently, regardless of their group membership, thus promoting fairness and equity in LLMs applied to medical settings.

Transparency in the development and deployment of LLMs is vital for maintaining public trust and fostering ethical use. Data privacy and security are of utmost importance, particularly when handling sensitive medical information. Compliance with regulations such as the Health Insurance Portability and Accountability Act and the General Data Protection Regulation is essential. Advanced privacy techniques like differential privacy can help protect patient data while ensuring useful statistical analysis. Moreover, secure data storage and transmission protocols should be in place to prevent unauthorized access and potential data breaches.

Developing a robust regulatory framework for LLMs in medicine is essential to ensure their safe and effective use. This framework should address issues related to the development, validation, and deployment of LLMs, as well as their ongoing maintenance and monitoring. Policymakers and regulatory agencies must work together to establish standards and guidelines that promote transparency, accountability, and responsible innovation without hindering progress. By considering ethical considerations, data privacy, and establishing a comprehensive regulatory framework, LLMs can be successfully integrated into medical practice in a manner that is both beneficial and responsible.

**Table 1:** Summary table of ten key suggestions for implementing LLMs in medicine

Ten Key Suggestions for LLMs in Medicine	
1.	Transfer learning, domain adaptation, few-shot learning, and zero-shot learning
2.	Reinforcement learning with expert feedback according to an explicit code of ethics
3.	Dynamic model with emphasis on more recent and more cited work
4.	Robust and specific evaluation metrics and benchmarks, along with clinical validation
5.	Data privacy, fairness-aware provisions, and diverse stakeholder involvement
6.	Inclusion of patients, caregivers, and other diverse perspectives
7.	Education and training prerequisites for the users
8.	A regulatory framework for their development, validation, deployment, maintenance, and monitoring
9.	Addressing cost and resource implications, exploring efficient models and cloud-based resources
10.	Ethical considerations, unbiased models, and mitigation strategies for potential risks and limitations

Conclusions

LLMs hold great promise for revolutionizing medical practice by improving diagnostic accuracy, predicting disease progression, and supporting clinical decision-making. The successful implementation of LLMs in medicine requires a multifaceted approach that addresses the unique challenges and considerations specific to the medical domain. Key aspects to consider include transfer learning, domain-specific fine-tuning, domain adaptation, reinforcement learning with expert input, dynamic training, interdisciplinary collaboration, education and training, evaluation metrics, clinical validation, ethical considerations, data privacy, and regulatory frameworks (Table 1). By addressing these essential factors, we can ensure that LLMs are developed, validated, and integrated into medical practice responsibly, effectively, and ethically. Furthermore, fostering an interdisciplinary and collaborative approach involving diverse perspectives will promote the creation of LLMs that address the needs of various medical disciplines and diverse patient populations. This comprehensive approach will help maximize the potential of LLMs to improve healthcare outcomes and transform the field of medicine. As we continue to explore the possibilities offered by LLMs, it is crucial to maintain a patient-centered focus, ensuring that the development and implementation of these technologies ultimately serve to enhance patient care and improve overall health outcomes for all.
